# The podoplanin-CLEC-2 interaction promotes platelet-mediated melanoma pulmonary metastasis

**DOI:** 10.1186/s12885-024-12194-w

**Published:** 2024-04-01

**Authors:** Minjia Sheng, Ran Sun, Jianxin Fu, Gao Lu

**Affiliations:** 1 Reproductive Medicine Center, China-Japan Union Hospital, Jilin University, Changchun, China; 2 Cardiovascular Biology Research Program, Oklahoma Medical Research Foundation, 73104 Oklahoma City, OK USA; 3 Central Research Laboratory, The First Affiliated Hospital of Soochow University, 188 Shizi Street, 215006 Suzhou, Jiangsu China

**Keywords:** Podoplanin, CLEC-2, Pulmonary metastasis, Platelet aggregation, Melanoma

## Abstract

**Background:**

Podoplanin (PDPN) expressed on tumour cells interacts with platelet C-type lectin-like receptor 2 (CLEC-2). This study aimed to investigate the role of the PDPN-platelet CLEC-2 interaction in melanoma pulmonary metastasis.

**Methods:**

Murine melanoma B16-F0 cells, which have two populations that express podoplanin, were sorted by FACS with anti-podoplanin staining to obtain purified PDPN + and PDPN- B16-F0 cells. C57BL/6J mice transplanted with CLEC-2-deficient bone marrow cells were used for in vivo experiments.

**Results:**

The in vivo data showed that the number of metastatic lung nodules in WT mice injected with PDPN + cells was significantly higher than that in WT mice injected with PDPN- cells and in WT or CLEC-2 KO mice injected with PDPN- cells. In addition, our results revealed that the platelet Syk-dependent signalling pathway contributed to platelet aggregation and melanoma metastasis.

**Conclusions:**

Our study indicates that the PDPN-CLEC-2 interaction promotes experimental pulmonary metastasis in a mouse melanoma model. Tumour cell-induced platelet aggregation mediated by the interaction between PDPN and CLEC-2 is a key factor in melanoma pulmonary metastasis.

**Supplementary Information:**

The online version contains supplementary material available at 10.1186/s12885-024-12194-w.

## Background

Melanoma is an aggressive and highly metastatic disease. It can spread to almost any area of the body and has rapid systemic dissemination. One of the most common sites for melanoma metastases is the lung. Metastatic melanoma develops when tumour cells dissociate from the primary lesion, migrate through the surrounding stroma, and invade blood vessels and/or lymphatics to form a tumour at a distant site [1]. However, the detailed mechanisms for melanoma haematogenous metastasis remain partly uncertain. In most cancers, the development of metastases requires that the cancer cells leave their primary site, circulate in the bloodstream, endure pressure in blood vessels, acclimate to new cellular surroundings at a secondary site, and escape deadly combat with immune cells [2, 3]. Tumour cell-induced platelet aggregation (TCIPA) plays an important role in tumour metastasis [4, 5].

Podoplanin (PDPN) is a mucin-type proteinwith a mass of36 to 43 kDa. It consists of a highly O-glycosylated extracellular domain, a transmembrane portion and a short cytoplasmic tail [5]. PDPN has a variety of functions, including regulating blood-lymphatic vessel development, cerebrovascular patterning and integrity, cell motility, tumorigenesis and metastasis [6, 7]. PDPN expressed on the surface of some tumour cells has been reported to contribute to cancer pathogenesis by promoting tumour cell invasion and spreading [8–10]. The Podoplanin (PDPN) is a transmembrane sialo-glycoprotein and its overexpression has been detected in many types of tumors, including squamous cell carcinoma, malignant mesothelioma, esophagus, Kaposi’s sarcoma, testicular seminoma and brain tumors, and is associated with poor clinical outcomes. The PDPN extracellular domain such as PLAG1, PLAG2, and PLAG3, are associated with tumor-induced platelet aggregation. O-glycosylated PLAG3 and PLAG4 interact with C-type lectin-like receptor 2 (CLEC-2), which is important for PDPN-induced platelet aggregation [10–15]. C-type lectin-like receptor 2 (CLEC-2) is a receptor for podoplanin that is expressed on the surface of platelets and facilitates tumour cell metastasis by inducing platelet activation, aggregation and the secretion of bioactive molecules, ultimately facilitating tumour cell survival, adhesion to the vessel wall, extravasation to new metastatic sites and growth [5, 12–18]. Toshiaki et al. used CLEC-2-deficient mice to show that CLEC-2 deficiency may suppress thrombus formation in tumour vessels in the lungs and subsequently inhibit systemic inflammation and cachexia [19]. Previous studies showed that the C-type lectin receptor CLEC-2 signals through a pathway that is critically dependent on the tyrosine kinase Syk, and support a model in which Syk and SLP76-dependent platelet activation through engagement of CLEC-2 by podoplanin. CLEC-2 initiates a Src- and Syk-dependent signaling cascade that is closely related to that of the 2 platelet ITAM receptors [20].

In our current study, we investigated the role of the PDPN-CLEC-2 interaction in melanoma pulmonary metastasis and its relationship with platelets. We used a podoplanin-expressing murine melanoma cell line and CLEC-2-deficient mice to establish a mouse model of experimental pulmonary metastatic melanoma.

## Materials and methods

### Cell line

The B16-F0 murine melanoma cell line was purchased from American Type Culture Collection (ATCC). Cells were cultured in Dulbecco’s modified Eagle’s medium (DMEM) containing 10% heat-inactivated foetal bovine serum (FBS), 1% GlutaMAX, and penicillin/streptomycin (Sigma) antibiotics at 37 °C in a humidified atmosphere of 5% CO_2_. Cells were sorted by FACS with Syrian hamster anti-podoplanin mAb (clone 8.1.1) staining. B16-F0 PDPN + cells and B16-F0 PDPN- cells were cultured under the same conditions described above. Mycoplasma were not detected in the cell culture.

### Mice

Eight- to twelve-week-old C57BL/6J mice (Jackson Laboratory) were housed under pathogen-free conditions. CLEC-2-deficient donor mice were generated in which exons 3 and 4 of the Clec-2 allele were flanked by loxP sites (CLEC-2 f/f). CLEC-2 f/f mice were crossed with Pf4Cre Tg mice (C57BL/6-Tg(Pf4-cre)Q3Rsko/J, Jackson Laboratory) to generate mice with CLEC-2-deficient platelets (CLEC-2 f/f; Pf4Cre mice). Compressed CO2 asphyxiation was used to sacrifice mice in accordance with the recommendations of the Panel on Euthanasia of the American Veterinary Medical Association. The study was approved by Ethics Committee of the Oklahoma Medical Research Foundation, all methods were carried out in accordance with relevant guidelines and regulations. This study was carried out in compliance with the ARRIVE guidelines 2.0.

### Flow cytometry

To measure podoplanin expression in B16-F0 cells, PDPN + and PDPN- cells were harvested by exposure to trypsin (Cellgro). After washing with Hank’s balanced salt solution (HBSS), cells were treated with Syrian hamster anti-podoplanin mAb (clone 8.1.1) or Syrian hamster IgG (Jackson ImmunoResearch) as a control for 20 min on ice. Then, the cells were incubated with goat anti-Syrian hamster fab2 DL488 antibody (Thermo) for 15 min on ice. To determine the CLEC-2 deletion efficiency in bone marrow-transplanted CLEC-2-deficient mice, whole blood was drawn from the mouse facial vein into EDTA-coated tubes. Five microlitres of whole blood was added to 95 µl of 0.1% bovine serum albumin (BSA) in HBSS and incubated with biotinylated anti-CLEC1B mAb (clone: 17D9, Abcam), biotinylated rat IgG 2b (BD Biosciences) and streptavidin-PE (BD Biosciences) for 30 min at room temperature. For platelet and tumour cell aggregation assays, platelets were incubated with 5 µM CellTracker Green (Invitrogen) for 10 min at 37 °C. Then, the washed platelets were mixed with tumour cells and incubated for 20 min at 37 °C. All flow cytometry experiments were carried out on a FACSCalibur (BD Biosciences). Data were collected and analysed using the CellQuest program (BD Biosciences).

### Western blotting

PDPN + and PDPN- B16-F0 cells were lysed with Tissue PE LB buffer (Bioscience) supplemented with complete proteinase inhibitor and phosphatase inhibitor cocktail (Roche Applied Science). The protein concentration was quantified using the Micro BCA protein assay kit (Pierce), and cell lysates containing 25 µg of total protein were resolved by 12% SDS‒PAGE. After blotting, the PVDF transfer membranes (Thermo) were probed with anti-podoplanin (Colne 8.1.1) and anti-GAPDH (Cell Signaling Technology) at a 1:1000 dilution, following incubation with the corresponding HRP-coupled secondary antibodies (Jackson ImmunoResearch) at a dilution of 1:5000. For Syk inhibitor experiments, 1.2 × 10^8^ isolated platelets from C57BL/6J mice were mixed with 6 × 10^5^ PDPN + or PDPN- B16-F0 cells in a volume of 200 µl for 1 min at 37 °C. R406 (10 µM, Selleck Chemicals) was used to pre-treat the platelets before incubation with tumour cells for 10 min at 37 °C to block Syk signalling. Then, the mixed samples were lysed and run on a 12% SDS‒PAGE gel. After blotting, the PVDF transfer membranes were probed with rabbit anti-Syk and rabbit anti-phospho-Syk antibodies (Cell Signalling, 1:1000 dilution) followed by goat anti-rabbit HRP antibody (Jackson ImmunoResearch). All blocking and antibody incubation steps were performed in 5% nonfat dry milk in PBS. ECL detection reagents were used for detection (Thermo).

### Cell proliferation assay

The in vitro growth of PDPN + and PDPN- B16-F0 cells was assessed using Cell Counting Kit-8 (Dojin Laboratories). A total of 1 × 10^3^ cells were seeded in 96-well plates and allowed to grow for 1 to 5 days. The cells were incubated with 10 µl of water-soluble tetrazolium salt-8 reagent for 1 h at 37 °C. Then, the optical density was measured at 450 nm using a microplate reader (POLARstar Omega BMG Labtech).

### Bone marrow engraftment

Male and female specific pathogen-free C57BL/6J mice (Jackson Laboratory), 4 to 5 weeks of age, were used as recipients. The mice were irradiated with 1100 cGy of ^137^Cs γ-rays 3 h before intravenous injection of bone marrow cells (4 × 10^6^) from both femurs and tibias of CLEC-2-deficient donor mice, and recipient mice were injected with bone marrow cells from wild-type donor mice as controls. Four to five weeks later, after determining the CLEC-2 deletion efficiency by flow cytometry, mice receiving bone marrow transplantation were used for the experiments.

### Histology

Lungs were fixed in 4% paraformaldehyde overnight at 4 °C and washed with PBS three times. Then, the lung tissues were processed into paraffin blocks, and 5 μm sections were cut. Specimens were deparaffinized and stained with haematoxylin and eosin (H&E). For immunofluorescence staining, cryosections (10 μm) of labelled tumour cell-injected lungs were air-dried and fixed with 4% paraformaldehyde for 10 min at RT, followed by blocking with 0.3% Triton-100, 3% donkey serum, and 3% BSA in PBS for 1 h at RT. The sections were incubated with the primary antibody (rat anti-CD41, BD Biosciences) for 2 h at RT. The secondary antibody used was donkey anti-rat DL649 (Jackson ImmunoResearch).

### Experimental lung metastasis assay

After reaching 70–80% confluence, PDPN + and PDPN- B16-F0 cells were harvested, washed and resuspended in HBSS. A total of 1 × 10^5^ PDPN + or PDPN- cells in a volume of 200 µl were inoculated intravenously into the lateral tail vein of bone marrow transplanted WT or CLEC-2 KO mice. After 14 days, the mice were euthanized, and the lungs were removed. The metastatic nodules on the surface of the lungs were counted under a dissection microscope (Nikon SMZ1500). The right lung tissues were fixed in 4% paraformaldehyde and embedded in paraffin blocks for H&E staining. Morphometric analyses were performed using ImageJ software. The metastatic foci in the cross-sections of the right lungs were counted, and the area covered by tumour masses was calculated relative to the total cross-sectional surface. For the Syk inhibitor assay, PDPN + and PDPN- B16-F0 cells were inoculated intravenously into WT mice via lateral tail vein injection. Mice were treated with 30 mg/kg R406 or DMSO twice a day for 3 days via intragastric feeding one day before injection. Fourteen days later, the number of metastatic nodules on the surface of the lungs was determined. For the short-term metastasis assay, B16-F0 PDPN + cells were labelled with CellTracker Orange (3 µM, Invitrogen), and PDPN- cells were labelled with CellTracker Green. The same number of PDPN + cells and PDPN- cells were mixed in HBSS at a concentration of 2.5 × 10^6^ cells/ml. WT and BMT CLEC-2 KO mice received 200 µl of mixed tumour cells (5 × 10^5^ cells/mouse) via tail vein injection. Mice were sacrificed 30 min, 2 h and 6 h after injection. The lungs were perfused with 50% OCT and sucrose freezing medium and embedded in OCT medium on dry ice. Cryo-cut sections of the lungs were stained with DAPI (Invitrogen). The tumour cells arrested in the lung were observed under a fluorescence microscope (Olympus). For the survival study, PDPN + cells and PDPN- cells (3 × 10^5^ cells/mouse) were inoculated intravenously into the lateral tail vein of WT mice. Mouse survival was checked daily for 30 days.

### Platelet and tumour cell aggregation assay

Whole blood was drawn from anaesthetized WT and CLEC-2 KO mice via the retroorbital plexus. Platelet-rich plasma was removed after two centrifugation steps (100 × g for 8 min), and a platelet pellet was generated after an additional centrifugation (1000 × g for 10 min). Platelets were resuspended in modified Tyrode’s buffer and labelled with CellTracker Green (5 µM) for 30 min at 37 °C. The number of platelets was determined by the MASCOT haematology system. PDPN + and PDPN- B16-F0 cells were collected in HBSS with calcium, and cell numbers were counted. Tumour cells were mixed with platelets at a final ratio of 1:200 (tumour cells: platelets). The samples were kept at 37 °C for 20 min and diluted with HBSS for flow cytometry analysis. The same samples were also stained with DAPI and observed under a fluorescence microscope. For the Syk inhibitor study, isolated platelets were incubated with 10 µM R406 (Syk inhibitor) for 10 min at 37 °C. Then, the platelets and tumour cells were subjected to flow cytometry and observed under a fluorescence microscope under the same conditions as those described above. For the in vivo platelet study, PDPN + cells and PDPN- cells (1 × 10^6^ cells/mouse) were inoculated intravenously into the lateral tail vein of WT mice. Before injection and 3 min after injection, whole blood was collected from the retro-orbital sinus, and the platelet number was determined with the Mascot haematology system to calculate the decrease.

### Tumorigenicity assay

PDPN + cells and PDPN- cells (1 × 10^5^ cells/mouse) in HBSS were inoculated subcutaneously into both sides of the backs of wild-type C57BL/6J mice (8–12 weeks). Two weeks later, the mice were euthanized, and the primary tumour tissues were harvested. Tumour weights and tumour volumes were determined. Tumour volume was calculated by the following formula: volume = W^2^ × L/2, where W = short diameter and L = long diameter.

### Statistical analysis

Statistical analysis was performed using Student’s *t* test after data were confirmed to fulfil the criteria of normal distribution and equal variance. Survival rates were compared using the long-rank test. Differences were considered statistically significant when *P* < 0.05. Data are expressed as the mean ± SEM. All data were analysed with Prism software (GraphPad).

## Results

### Podoplanin expression in different populations of the B16 murine melanoma cell line

**Fig. 1:**
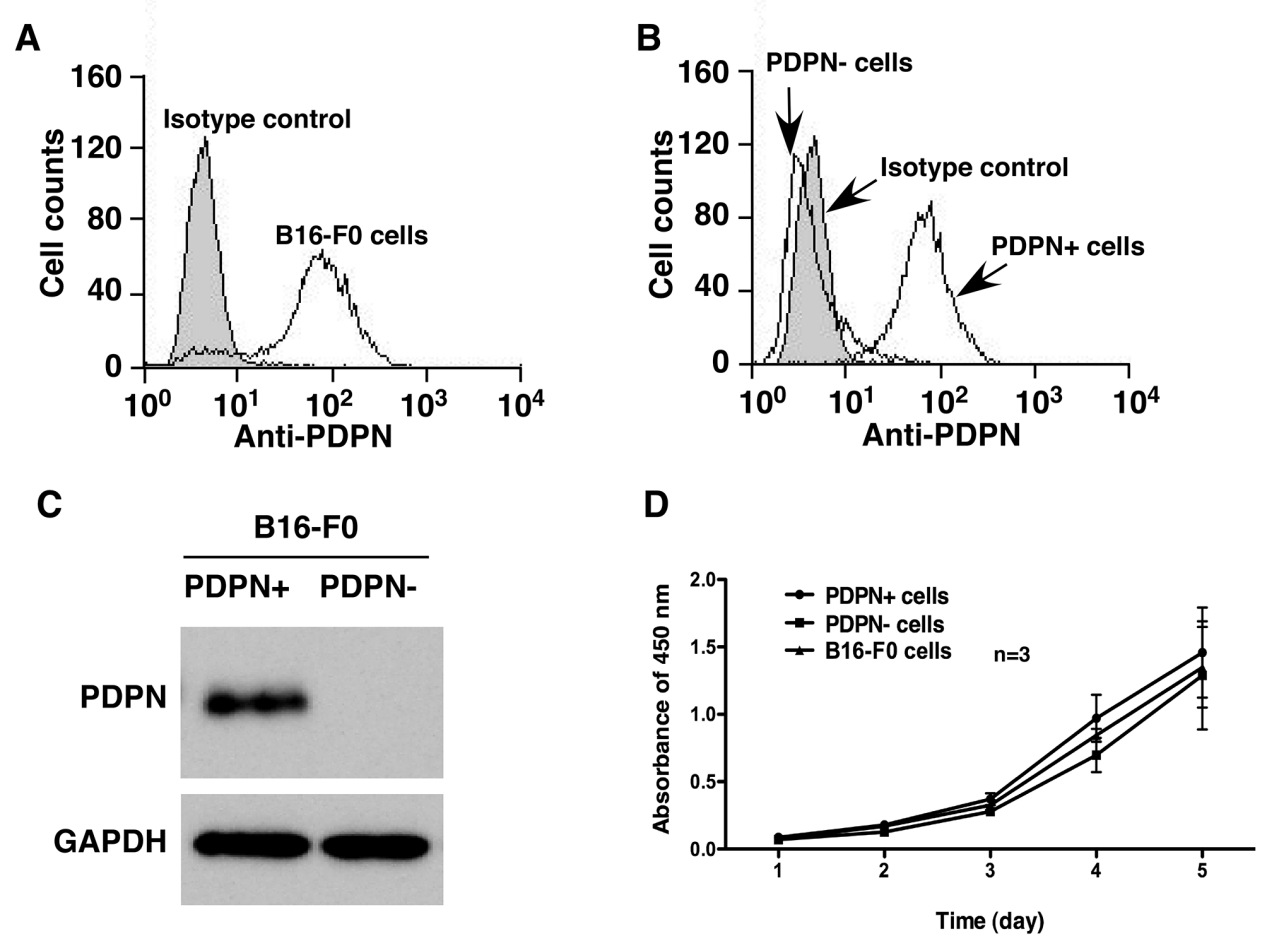
Podoplanin expression in the B16 melanoma cell line. **(A).** FACS analysis of podoplanin expression in B16-F0 cells. (**B).** FACS analysis of podoplanin expression in PDPN + and PDPN- B16-F0 cells. (**C).** Western blot detection of podoplanin expression in the cell lysates of PDPN + B16-F0 cells and PDPN- B16-F0 cells. (**D).** Cell growth speed evaluated by Cell Counting Kit assay. Data shown are the mean results ± SEMs of a representative experiment performed in triplicate (*n* = 3)

We used the murine melanoma cell line B16-F0, which has two populations with differential podoplanin expression, to perform the experiments (Fig. 1, A and B). B16-F0 cells were sorted by FACS with anti-podoplanin staining. We thus established PDPN + and PDPN- B16-F0 murine melanoma cells (Fig. 1, B and C). Using two populations of cells sorted from one cell line can avoid individual differences from the experimental cells. We cultured the two cell lines in vitro and found that the growth speed of these two populations of cells in vitro was not significantly different (Fig. 1, D). We also evaluated the growth of PDPN + and PDPN- cells when these two cell lines were inoculated subcutaneously. As shown in Supplemental Fig. 1, there were no differences in the size or volume of the subcutaneous tumours in vivo. H&E staining of the two types of subcutaneous tumours showed similar histology.

### The Podoplanin-CLEC-2 interaction promotes melanoma pulmonary metastasis

**Fig. 2:**
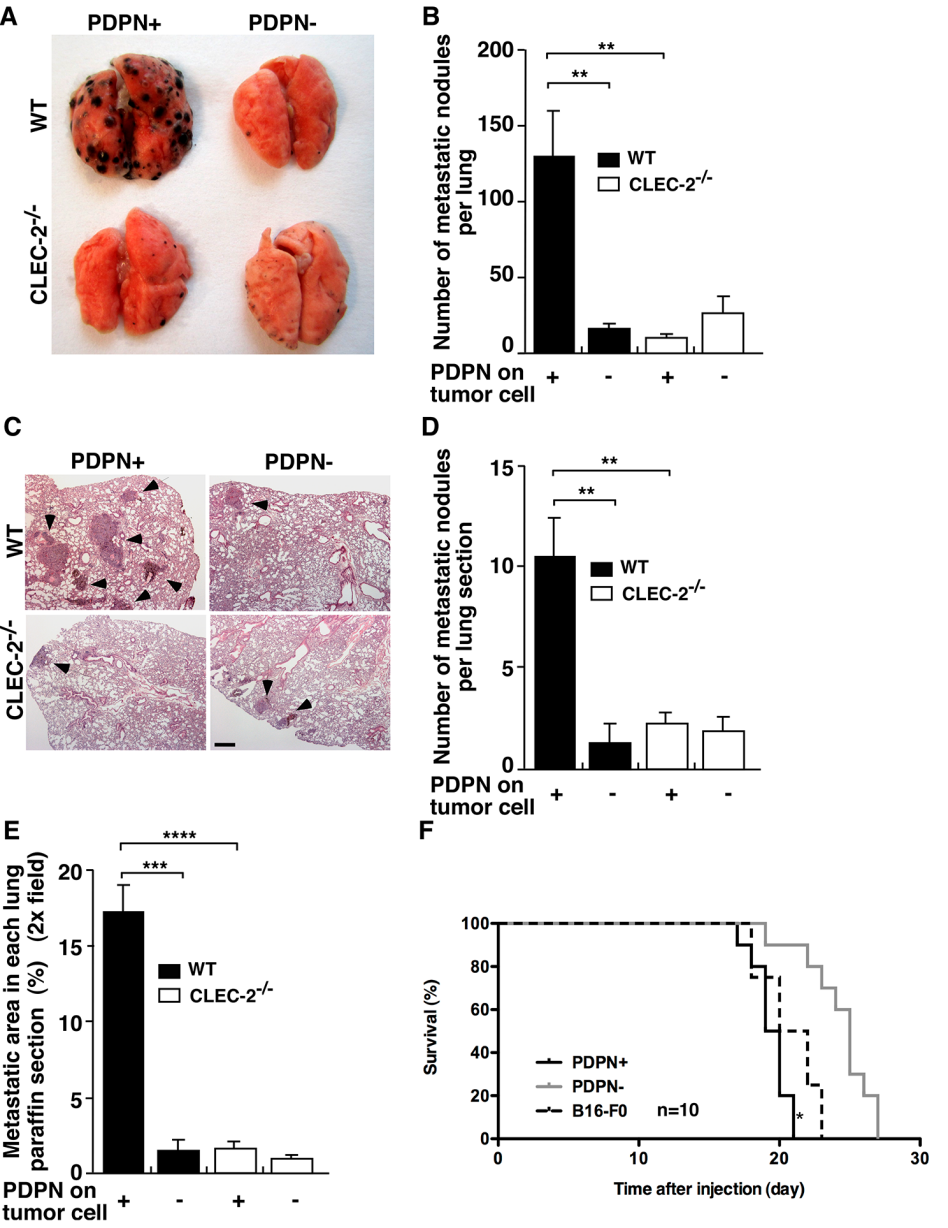
The podoplanin-CLEC-2 interaction promotes melanoma pulmonary metastasis and decreases mouse survival *in vivo*. PDPN + or PDPN- B16-F0 cells (1 × 10^5^) were injected intravenously into bone marrow transplant (BMT) WT and CLEC-2 KO mice via the tail vein. (**A).** Representative gross images of the metastatic lung nodules from the four groups (PDPN + and PDPN- cells injected individually into BMT WT mice and BMT CLEC-2 KO mice) are shown. (**B).** The numbers of metastatic nodules on the surface of the lung were analysed. Data are means ± SEMs. (**C).** Representative cross-sections of H&E-stained lung tissues 14 days after tumour cell injection. Arrows point to metastatic foci. Scale bar = 500 μm. (**D).** The numbers of metastatic nodules in each right lung section. Data are means ± SEMs. (**P < 0.01) (**E).** Graph showing the area covered by the tumour mass relative to the total lung area in the cross-section determined by ImageJ. Data are means ± SEMs. (***P < 0.001, ****P < 0.0001) (**F).** Survival rates of mice injected with PDPN + cells and PDPN- cells over time. (*P < 0.0001 by log-rank test)

A syngeneic model of experimental metastasis was used to assess the role of the podoplanin-CLEC-2 interaction in melanoma metastasis *in vivo.* We intravenously inoculated PDPN + and PDPN- B16-F0 cells into WT C57BL/6J mice or WT mice transplanted with CLEC-2-deficient bone marrow (CLEC-2 KO) via lateral tail vein injection. CLEC-2 deletion efficiency after bone marrow transplantation was examined by flow cytometry (sup. Figure 2). The results showed that the number of metastatic lung nodules in the WT mice injected with PDPN + cells was significantly higher than that in WT mice injected with PDPN- cells and in WT or CLEC-2 KO mice injected with PDPN- cells (Fig. 2, A and B). Suppression of metastatic tumours was observed in the lungs of WT mice injected with PDPN- cells, CLEC-2 KO mice injected with PDPN + cells and WT mice injected with PDPN- cells by H&E staining (Fig. 2, C). Both the metastatic nodule number and metastatic area in WT mice injected with PDPN + cells were higher than those in the other three groups (Fig. 2, D and E). We also evaluated the survival of the mice after intravenous injection of PDPN + cells and PDPN- cells. The data showed that mice injected with PDPN + cells had a significantly poorer survival rate than mice injected with PDPN- cells (Fig. 2F).

### Podoplanin and CLEC-2 affect the melanoma cell association with platelets in vitro

**Fig. 3:**
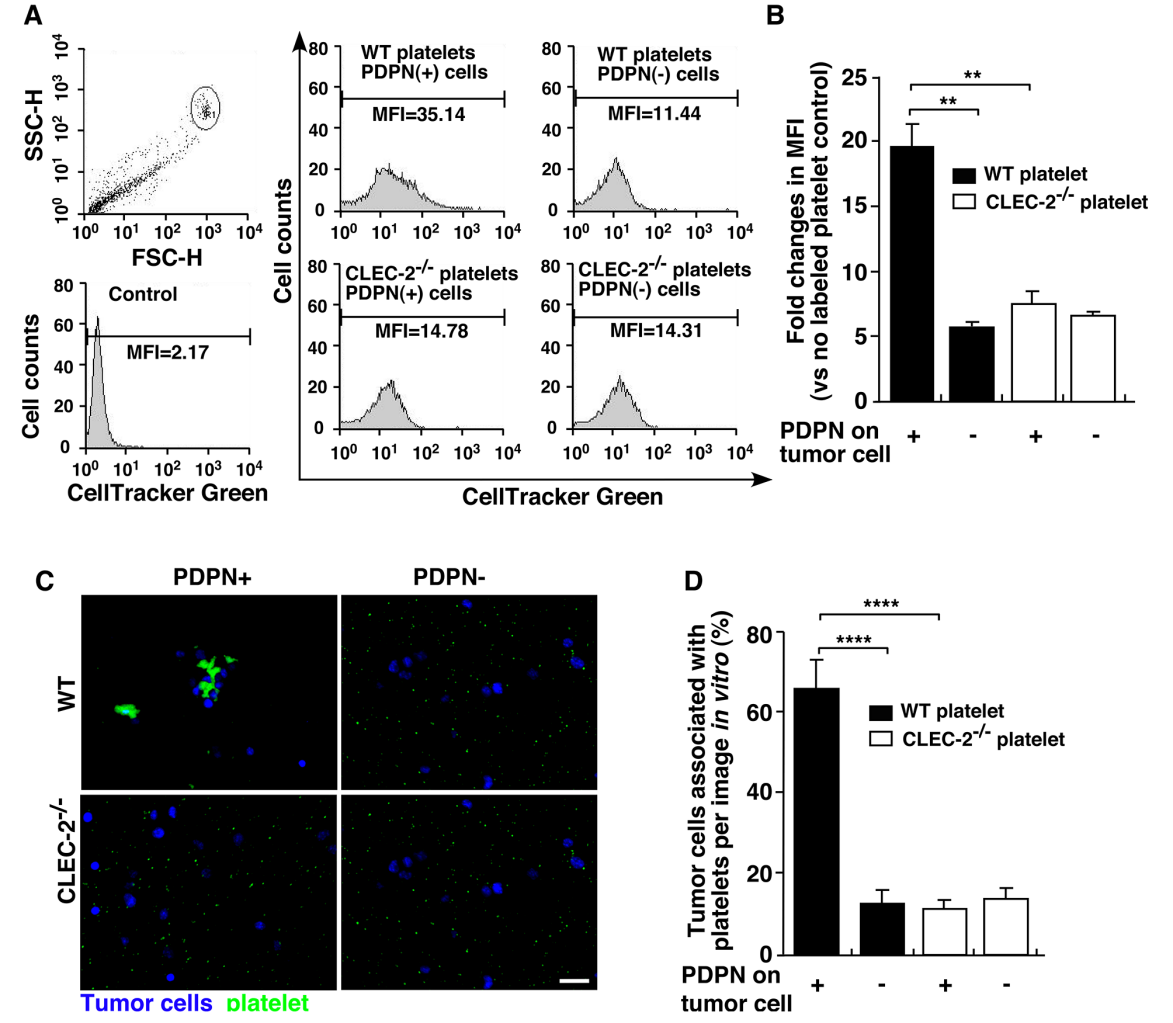
The podoplanin-CLEC-2 interaction increases platelet and melanoma tumour cell aggregation *in vitro.* PDPN + and PDPN- B16-F0 cells were mixed with platelets from WT or CLEC-2 KO mice in vitro to observe the interaction between the tumour cells and platelets. (**A).** Flow cytometry profiles of platelets from WT and CLEC-2 KO mice mixed with PDPN + or PDPN- B16-F0 tumour cells. The mean fluorescence intensity (MFI) of each group is shown. The MFI of platelets without CellTracker labelling was used as a control. (**B).** Fold changes in mean fluorescence intensity compared with the control (unlabelled WT platelets mixed with PDPN + cells) were determined. Data are means ± SEMs (*n* = 3). (**P < 0.01) (**C).** Representative immunofluorescence images of tumour cells mixed with platelets in vitro. WT platelets mixed with PDPN + cells induced platelet and tumour cell aggregation. Scale bar = 10 μm. (**D).** The numbers of tumour cells associated with platelets were quantified by evaluating sections. At least 6 independent images for each sample were analysed. Data are means ± SEMs. (****P < 0.0001)

PDPN + and PDPN- cells were mixed with WT and CLEC-2 KO platelets and incubated *in vitro*. The flow cytometry profiles showed that WT platelets mixed with PDPN + cells had a higher mean fluorescence intensity than the other three groups (Fig. 3, A and B). We detected DAPI-stained tumour cells and CellTracker Green-labelled platelet aggregates under a fluorescence microscope. Consistent with the flow cytometry profiles, the results of this study showed that more WT platelets were associated with PDPN + tumour cells than was observed with the other three groups (Fig. 3, C). The association of melanoma cells with platelets was determined by counting the number of tumour cells under a microscope (Fig. 3, D). A total of 64.96% of the tumour cells were associated with platelets in the mixture of WT platelets with PDPN + cells, which was significantly higher than in the WT platelets with PDPN- cells (12.00%, *p* < 0.001), CLEC-2 KO platelets with PDPN + cells (10.96%, *p* < 0.001) and CLEC-2 KO platelets with PDPN- cells (12.90%, *p* < 0.001) groups.

### The podoplanin-CLEC-2 interaction induces platelet activation and promotes melanoma cell metastasis in a short-term pulmonary metastatic assay

**Fig. 4:**
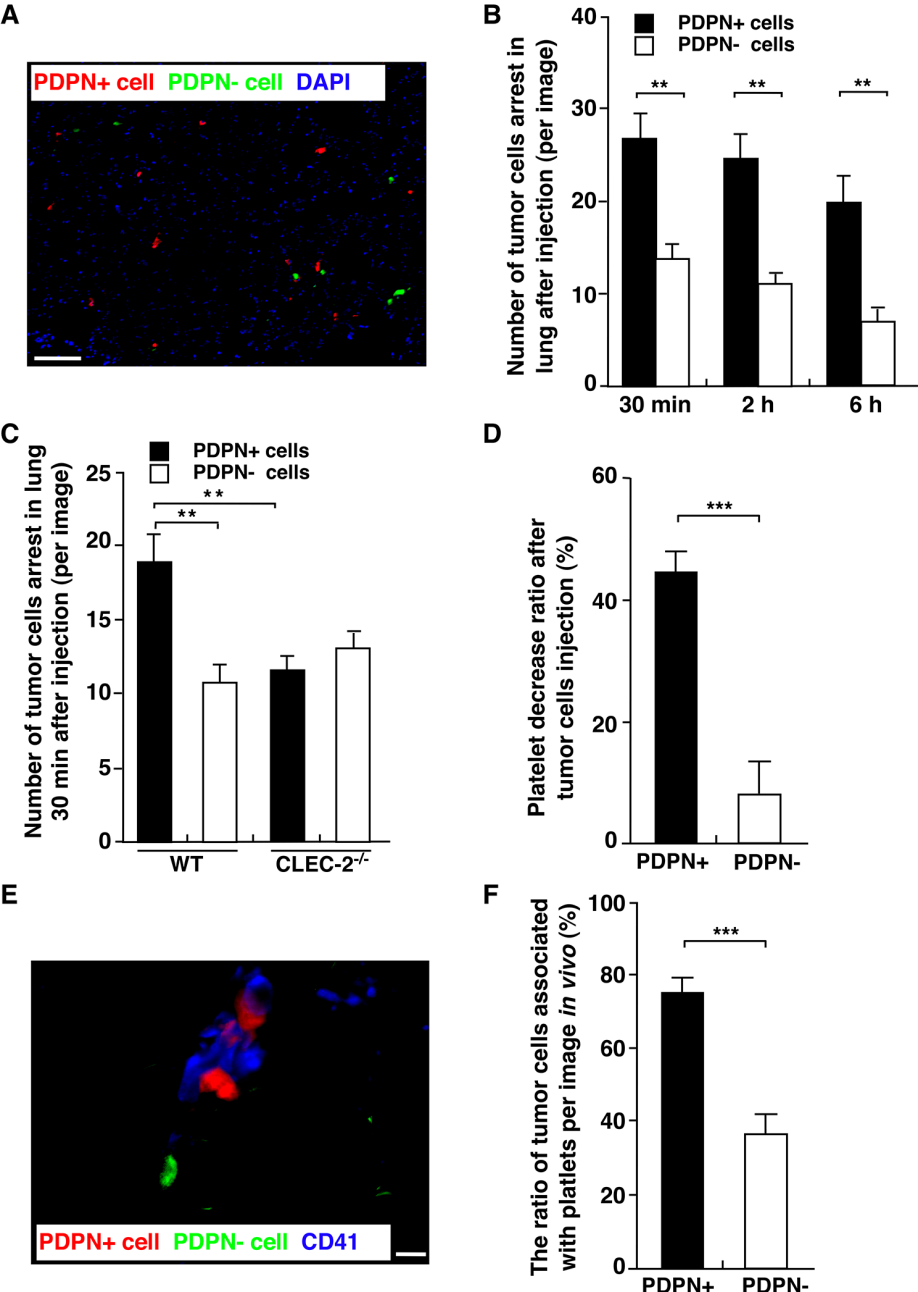
Podoplanin expression facilitates melanoma tumour cell arrest in the lung through its interaction with platelets *in vivo.* CellTracker-labelled mixed PDPN+ (red) and PDPN- (green) B16-F0 cells were injected intravenously into WT mice. Mice were sacrificed at different time points (30 min, 2 h, 6 h), and the lungs were removed. **(A).** Representative image of labelled PDPN + and PDPN- cells that were arrested in the lung 2 h after cell injection. Scale bar = 50 μm. **(B).** The average numbers of labelled tumour cells per microscopic field were determined in cryotome-cut lung sections at each time point. At least 6 images for each lung were analysed. (**P < 0.01) **(C).** The average number of labelled cells in WT mice and CLEC-2 KO mice 30 min after injection. (**P < 0.01) **(D).** Graph showing the ratio of the decrease in platelet number (determined with the MASCOT haematology system) 3 min after injection of tumour cells compared to the platelet number before injection. Six mouse blood samples from each group were analysed. (***P < 0.001) **(E).** Cryosections of the lungs were stained for platelets with an anti-CD41 antibody. **(F).** The numbers of labelled PDPN + and PDPN- cells associated with platelets determined by evaluating the sections. Seven images for each section were analysed. Data are means ± SEMs. (***P < 0.001)

Quantification of the labelled tumour cells in lung cryosections at different time points after injection showed that the number of PDPN + cells was significantly higher than the number of PDPN- cells at all time points (Fig. 4, A and B). Similarly, the number of PDPN + cells in WT mouse lungs was higher than that in the other three groups. There was no difference between the PDPN- cell injection groups and the CLEC-2 KO mouse groups (Fig. 4, C). To determine tumour cell-induced platelet aggregation in vivo, lung cryosections were stained with the platelet marker CD41. There were more PDPN + cells (red) covered with platelets than PDPN- cells (green) covered with platelets. The data showed that PDPN + cells associated with platelets were significantly more abundant than PDPN- cells associated with platelets (Fig. 4, E and F). Whole blood was collected from the retro-orbital sinus before PDPN + and PDPN- tumour cell injection and 3 min after injection. The data showed that the decrease in platelet percentage among whole blood cells after PDPN + cell injection was significantly larger than that after PDPN- cell injection (Fig. 4, D).

### The syk-dependent signalling pathway plays a role in platelet aggregation and melanoma metastasis

**Fig. 5:**
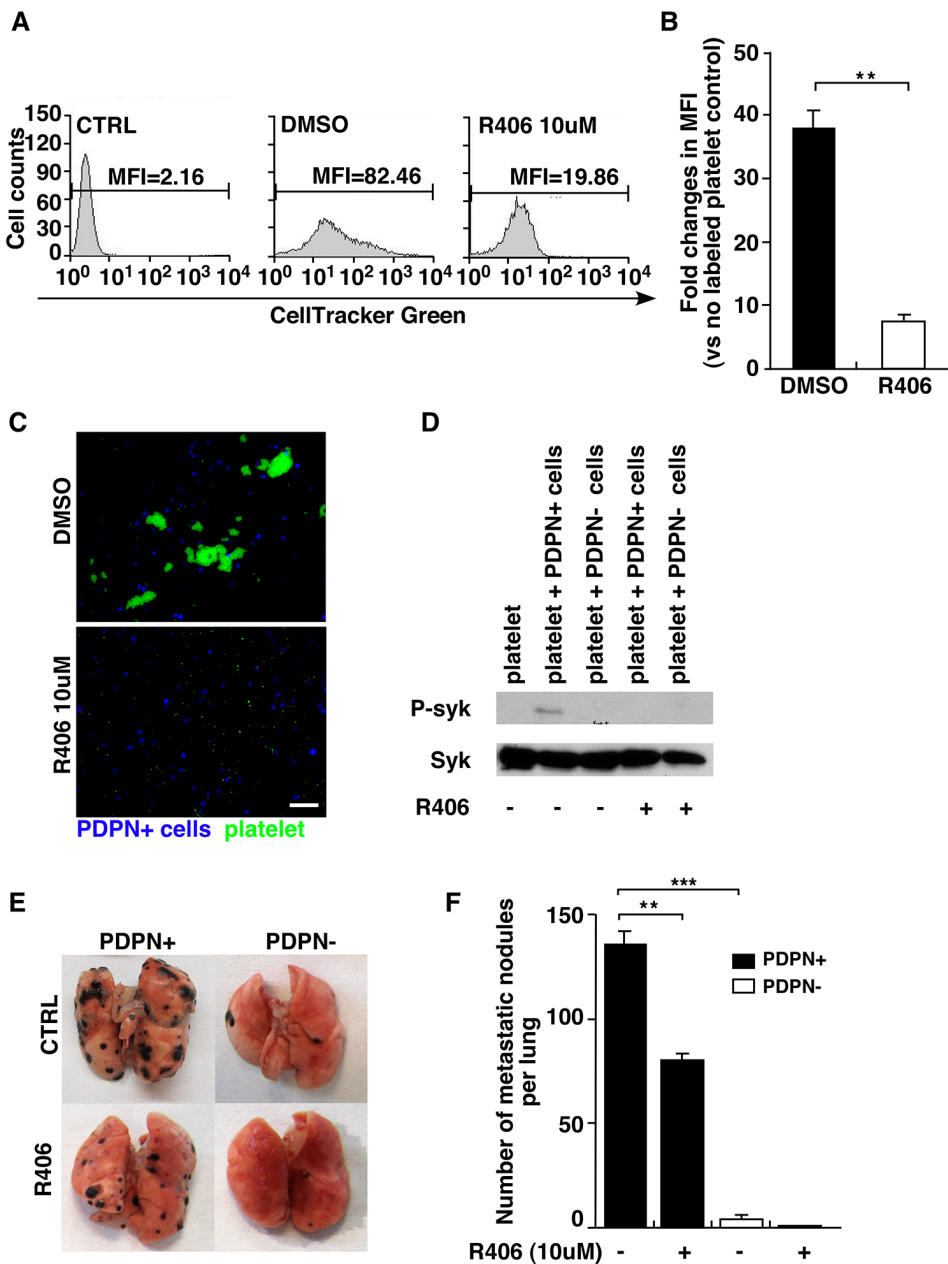
A Syk inhibitor blocks podoplanin-expressing melanoma tumour cell and platelet aggregation in vitro and pulmonary metastasis *in vivo.* (**A).** Syk inhibitor (R406)- or DMSO-pretreated platelets were incubated with PDPN + cells, and the mean fluorescence intensity (MFI) of each group was measured by flow cytometry. The MFI of platelets without CellTracker labelling with PDPN + cells was used as a control. The MFI of each group is shown. (**B).** Fold changes in mean fluorescence intensity compared with the control were determined. Data are means ± SEMs (*n* = 3) (**P < 0.01) (**C).** Representative immunofluorescence images of PDPN + tumour cells mixed with platelets pretreated with DMSO or R406 in vitro. Scale bar = 10 μm. (**D).** Western blot profiles showing phospho-syk expression after tumour cells were mixed with platelets. (**E).** Images of metastatic lung nodules from tumour cell-inoculated mice treated with 30 mg/kg R406 or DMSO. (**F).** The number of metastatic nodules on the surface of the lung was analysed. Data shown are the means ± SEMs (*n* = 3). (**P < 0.01) (***P < 0.001)

PDPN binding to CLEC-2 induces Syk-dependent signalling in platelets. We used the Syk inhibitor R406 to block platelet binding to tumour cells to assess the role of Syk in the PDPN-CLEC-2 interaction. The flow cytometry profiles showed that the MFI in the R406-treated group was lower than that in the DMSO group (Fig. 5, A and B). We also used fluorescence microscopy to observe DAPI-stained tumour cells and CellTracker Green-labelled platelet aggregates after treatment with the Syk inhibitor or DMSO. The results showed that the inhibitor R406 blocked the aggregation of platelets and tumour cells (Fig. 5, C). PDPN + cells and PDPN- cells were mixed with platelets that had been pretreated with R406 or DMSO. Cell lysates were collected after the interaction. We performed western blotting to detect Syk and phospho-Syk expression in each group (Fig. 5, D). Only PDPN + cells mixed with platelets without the inhibitor R406 expressed phospho-Syk, which indicates that the podoplanin-CLEC-2 interaction activates Syk. We intravenously inoculated PDPN + and PDPN- cells into WT mice. Mice were treated with 30 mg/kg R406 or DMSO twice per day for 3 days via intragastric feeding before injection. R406 inhibited lung metastasis to some extent in the PDPN + cell injection group (Fig. 5, E and F).

## Discussion

Metastasis is the major cause of death from most cancers, including melanoma. Tumour cell-platelet interactions have been suspected to play a role in tumour metastasis [21]. Podoplanin on tumour cells has been shown to promote pulmonary metastasis by inducing platelet aggregation [22, 23]. However, how podoplanin contributes to tumour metastasis is still unclear. CLEC-2 on the surface of platelets was identified as a receptor for podoplanin and induces platelet aggregation in the tumour metastasis process [24]. In our experiments, we used the B16 melanoma cell line to demonstrate the role of podoplanin in melanoma pulmonary metastasis because B16 cells express podoplanin, which is involved in platelet aggregation [25]. We discovered that the podoplanin-CLEC-2 interaction promoted experimental melanoma pulmonary metastasis through platelet and tumour cell aggregation. Pulmonary metastasis and platelet aggregation decreased in the absence of either podoplanin or CLEC-2. Our results show that a Syk inhibitor can partially block platelet aggregation and pulmonary metastasis in vitro and in vivo, which demonstrates that the syk-dependent signalling pathway in the podoplanin-CLEC-2 interaction plays a role in platelet aggregation and melanoma pulmonary metastasis.

Melanoma metastasis occurs in the early stage of the disease, and melanoma cells spread mainly through haematogenous dissemination. Studying how melanoma cells survive in the bloodstream is key to preventing melanoma metastasis. From the in vitro platelet and tumour cell aggregation assay, we found that the podoplanin-CLEC-2 interaction promoted the aggregation of platelets and melanoma cells. The aggregation of platelets and tumour cells protects tumour cells from immune elimination and promotes their arrest on the endothelium, supporting tumour cell dissemination [26–28]. Additionally, platelets release soluble factors such as PDGF, TGF-β, and ADP after aggregation, which changes the microenvironment to be suitable for tumour metastasis [29, 30]. In our experiment, platelets and melanoma cells aggregated in vitro only when podoplanin and CLEC-2 were present at the same time. This finding possibly indicates that the interaction between podoplanin and CLEC-2 promotes pulmonary metastasis by activating platelet and melanoma cell aggregation. In a previously reported experimental model of metastasis, platelet knockout mice showed marked protection against metastasis. Furthermore, some studies have shown that experimental blockade of platelet adhesion receptors, such as glycoprotein Ib-IX-V (GPIb-IX-V), integrin αIIbβ3 and P-selectin, can inhibit tumour cell-induced platelet aggregation and diminish metastasis in experimental mouse models [31, 32]. Xu et al. used the human podoplanin antibody SZ168 to block the growth and pulmonary metastasis of human malignant melanoma by inhibiting the interaction between tumour PDPN and platelet CLEC-2 [13]. In our experiment, either a lack of podoplanin on melanoma cells or CLEC-2 depletion on platelets blocked platelet aggregation and reduced melanoma pulmonary metastasis in vivo. Our results demonstrate that the podoplanin-CLEC-2 interaction activates platelets and promotes the aggregation of platelets and melanoma cells. Then, the activated platelets facilitate the formation of metastatic melanoma cell foci in the lung. Conversely, this suggests that inhibition of platelet aggregation via depletion of podoplanin and CLEC-2 expression may diminish melanoma pulmonary metastasis.

**Fig. 6:**
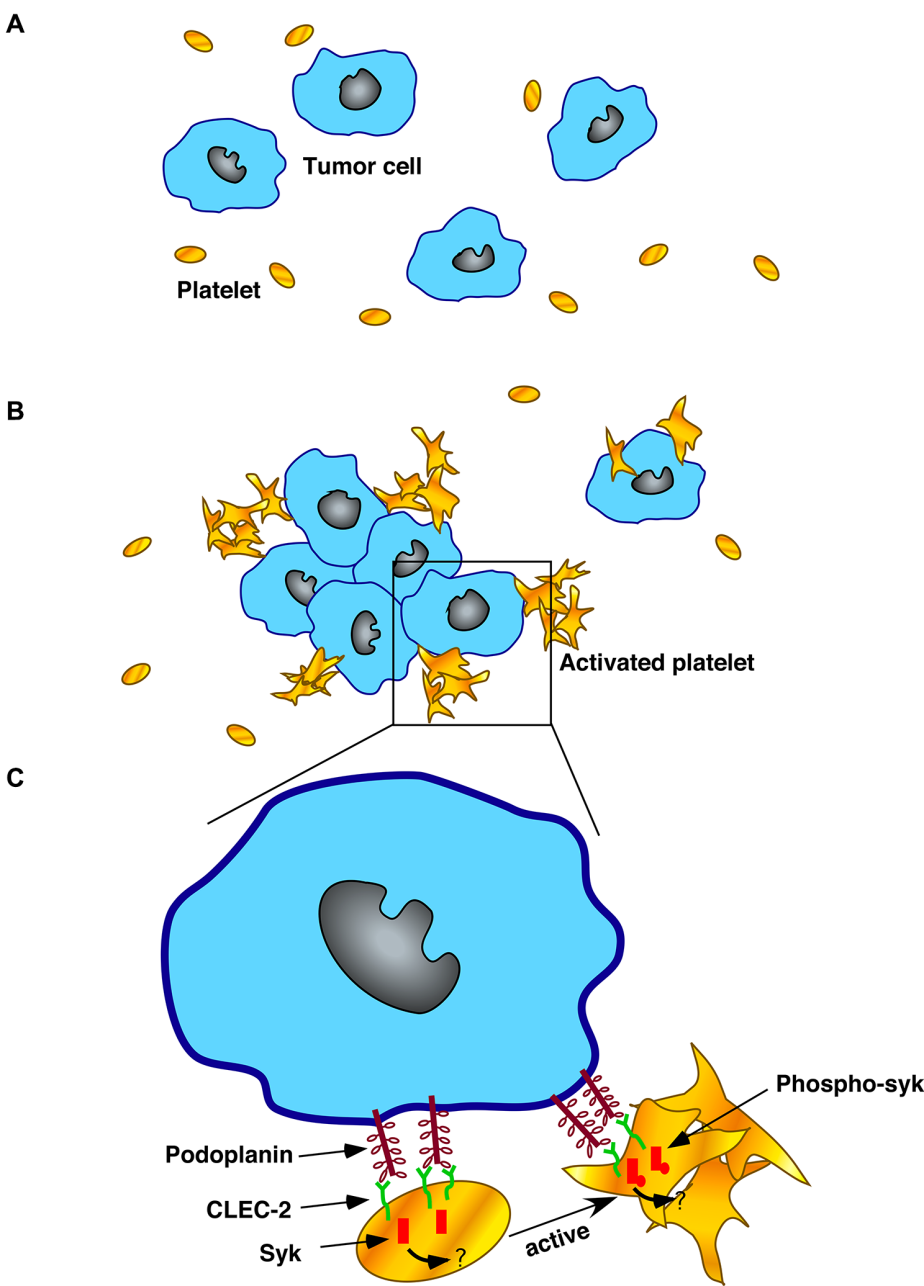
The role of Syk in the podoplanin-CLEC-2 interaction in the aggregation of melanoma tumour cells and platelets. **(A-B).** Podoplanin on the surface of tumour cells interacts with CLEC-2 on the platelet membrane, leading to the activation of platelets and inducing platelet and tumour cell aggregation. **(C).** Podoplanin on the surface of tumour cells interacts with CLEC-2 on the surface of platelets, inducing the phosphorylation of syk on the CLEC-2 cytoplasmic tail domain. The activated platelets aggregated and induced tumour cell aggregation as well. However, the mechanism of syk involvement remains unclear

CLEC-2 induces platelet activation through Syk tyrosine kinases [33]. The phosphorylation of Syk tyrosine kinases will initiate the signalling cascade and activate platelets [34, 35]. When podoplanin on tumour cells interacts with CLEC-2 on platelets, Syk is phosphorylated by the interaction of the YxxL sequence on the CLEC-2 cytosolic tail with the SH2 tandem domains on Syk [36–38]. Murine platelets deficient in Syk failed to respond to rhodocytin, suggesting that Syk is crucial for CLEC-2-mediated signal transduction [39]. In our study, Syk was phosphorylated when WT platelets were mixed with PDPN + cells *in vitro.* Additionally, a Syk inhibitor blocked the activation of Syk in PDPN + cells. Our results showed that a Syk inhibitor can suppress platelet and tumour cell aggregation in vitro and partially inhibit pulmonary metastasis in vivo. However, the inhibition of pulmonary metastasis with a Syk inhibitor in vivo was not complete, which means that the Syk-dependent signalling pathway is not the only pathway that affects the podoplanin-CLEC-2 interaction in vivo. The Syk-dependent signalling pathway is a novel signalling pathway in platelets that activates CLEC-2 on the cell surface [30]. Podoplanin on tumour cells activates the receptor CLEC-2, which recruits Syk to phosphorylate tyrosine in CLEC-2. This complex probably activates Syk through both conventional ITAM-mediated Syk activation pathways and by binding of the integrin β-chain to the amino-terminal SH2 domain of Syk. Syk activation leads to platelet activation through SH2 domain-containing leukocyte protein 76 (SLP76) and PLCγ2 [40]. Above all, the role of the podoplanin-CLEC-2 interaction in experimental melanoma pulmonary metastasis partially depends on the Syk signalling pathway. However, the detailed mechanism of the Syk-dependent signalling pathway in the podoplanin-CLEC-2 interaction still needs to be further studied (Fig. 6).

In conclusion, our present study shows that the podoplanin-CLEC-2 interaction promotes experimental pulmonary metastasis in a mouse melanoma model. The podoplanin-CLEC-2 interaction induces the aggregation of platelets and melanoma cells and facilitates tumour cell spreading to remote organs *in vivo.* Our data indicate that PDPN/CLEC-2-mediated tumour cell-induced platelet aggregation is a key factor for melanoma pulmonary metastasis. Based on these studies, podoplanin and CLEC-2 may be predictors of tumour metastasis and targets for future antimetastatic therapy.

### Electronic supplementary material

Below is the link to the electronic supplementary material.

Supplementary Material 1

Supplementary Material 2

## Data Availability

All data and materials are presented in the manuscript, which will be freely available to any scientists wishing to use them for non-commercial purposes.

## Declarations

### Animal studies

The animal studies were conducted according to protocols approved by the Institutional Animal Care and Use Committee of the Oklahoma Medical Research Foundation.

## Abbreviations

PDPN: Podoplanin
CLEC-2: C-type lectin-like receptor 2
TCIPA: Tumour cell-induced platelet aggregation
Syk: Src family kinases
H&E: Haematoxylin and eosin
HBSS: Hank’s balanced salt solution
PBS: Phosphate buffered saline
mAb: Monoclonal antibody
FACS: Fluorescence activated cell sorting
BSA: Bovine serum albumin
WT: Wild Type
