# Supplementary material for: The podoplanin-CLEC-2 interaction promotes platelet-mediated melanoma pulmonary metastasis
Source: BMC Cancer. 2024 Apr 1;24:399. doi: 10.1186/s12885-024-12194-w (PMC10983743; doi:10.1186/s12885-024-12194-w)
Supplement: Supplementary file 1 — Supplementary Material 1 [file 12885_2024_12194_MOESM1_ESM.pdf]

Figure 1 Panel C

Original image for western blot

Cropped image for western blot in the manuscript

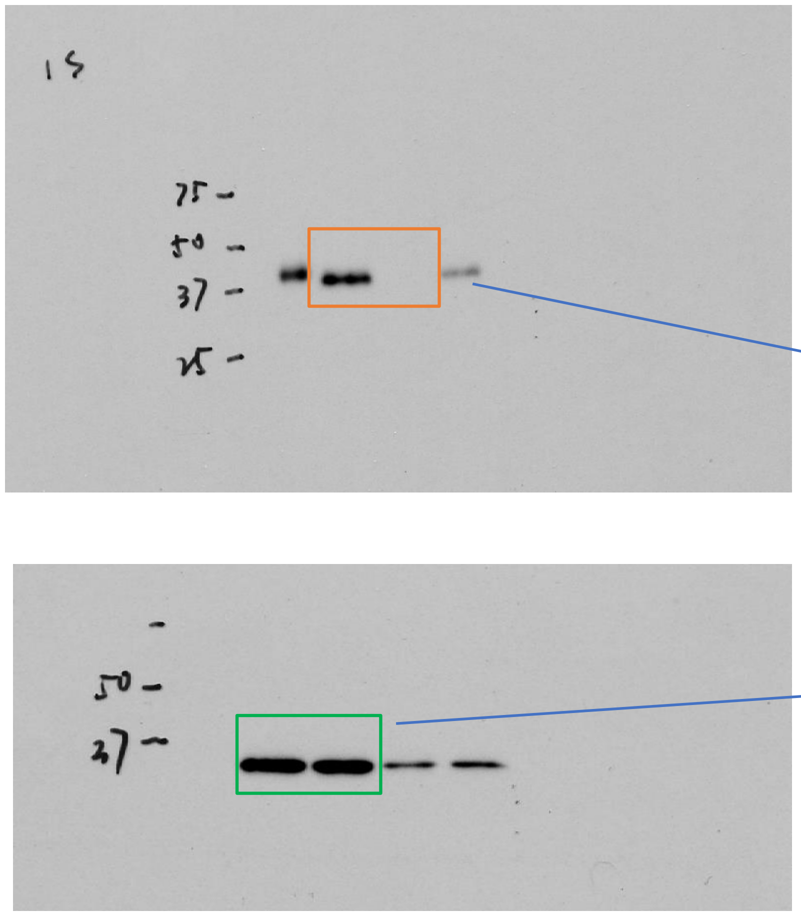


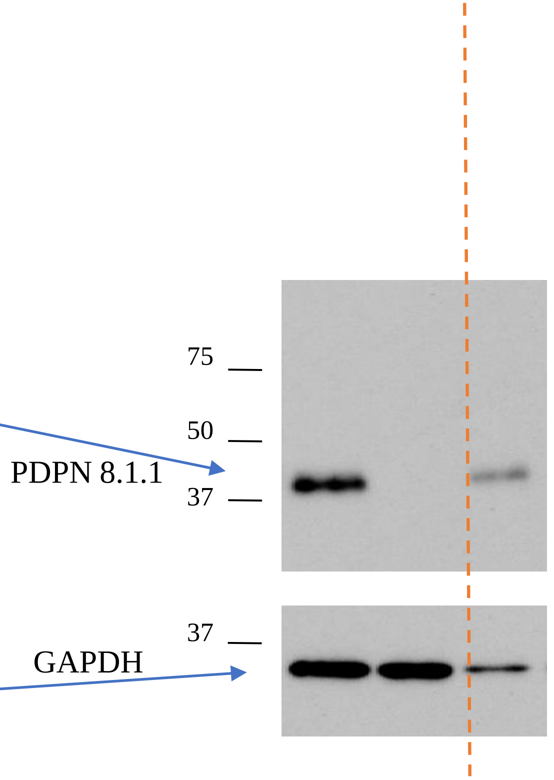


The image in the right of red line was cropped

Western blot Film For
Figure 1 Panel C

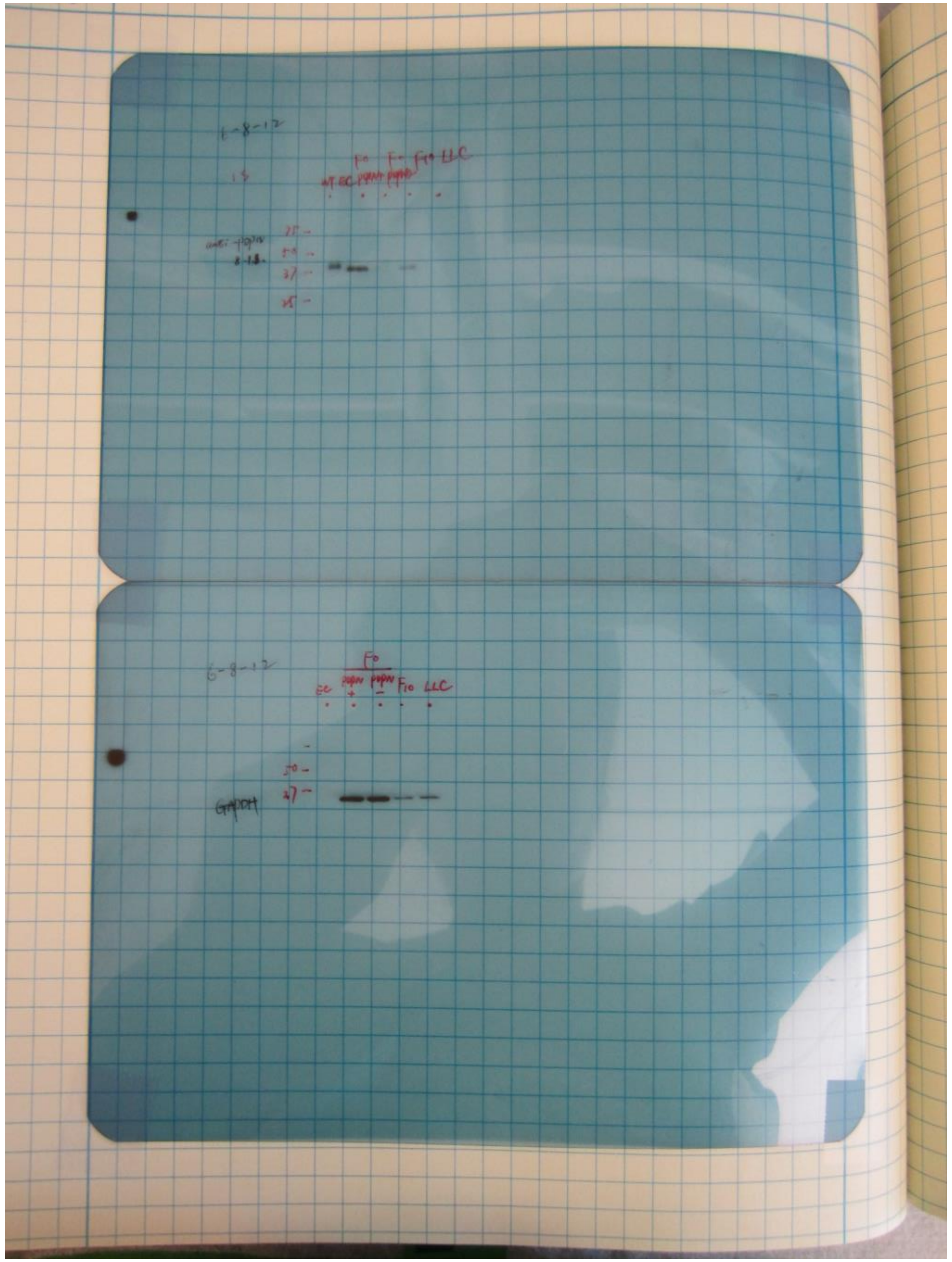

Figure 5 Panel D

Original image for western blot

Cropped image for western blot in the manuscript

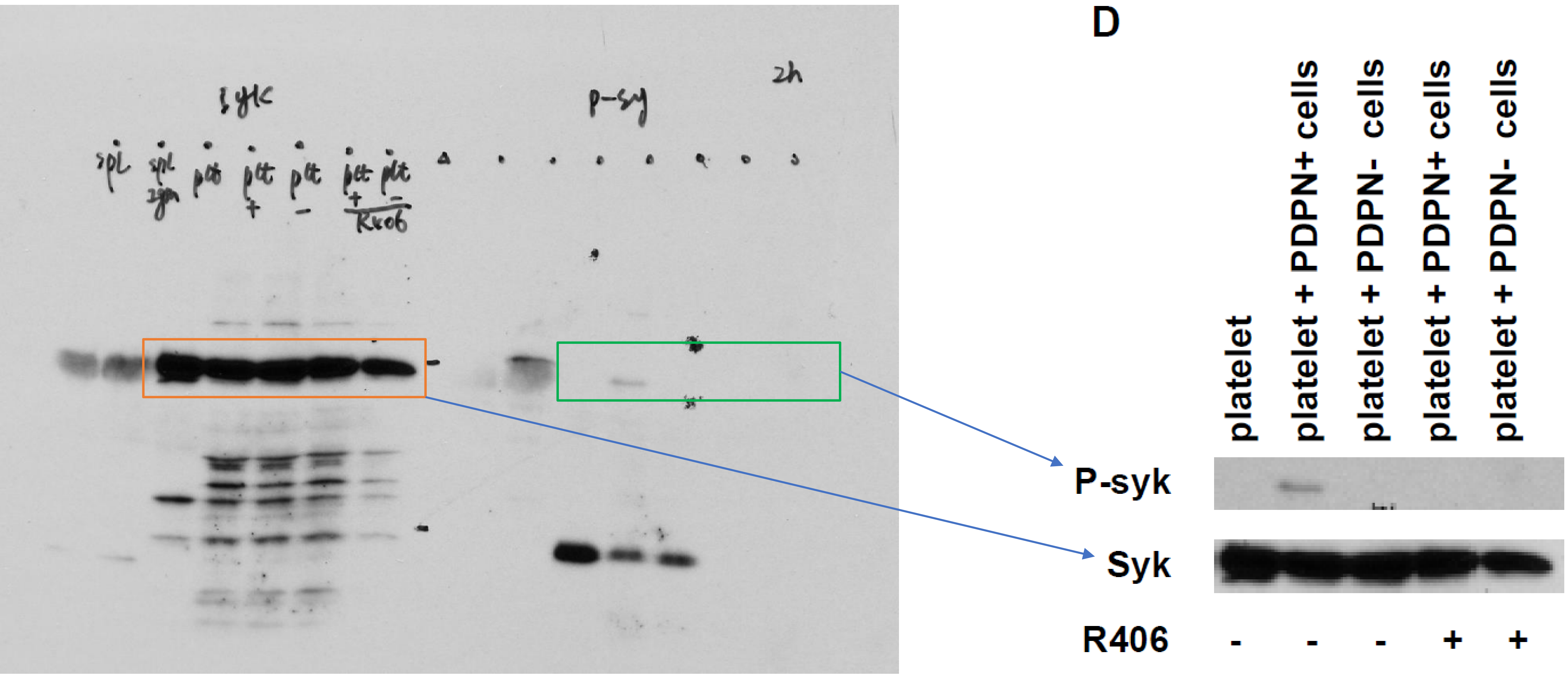

Western blot Film For
Figure 5 Panel D

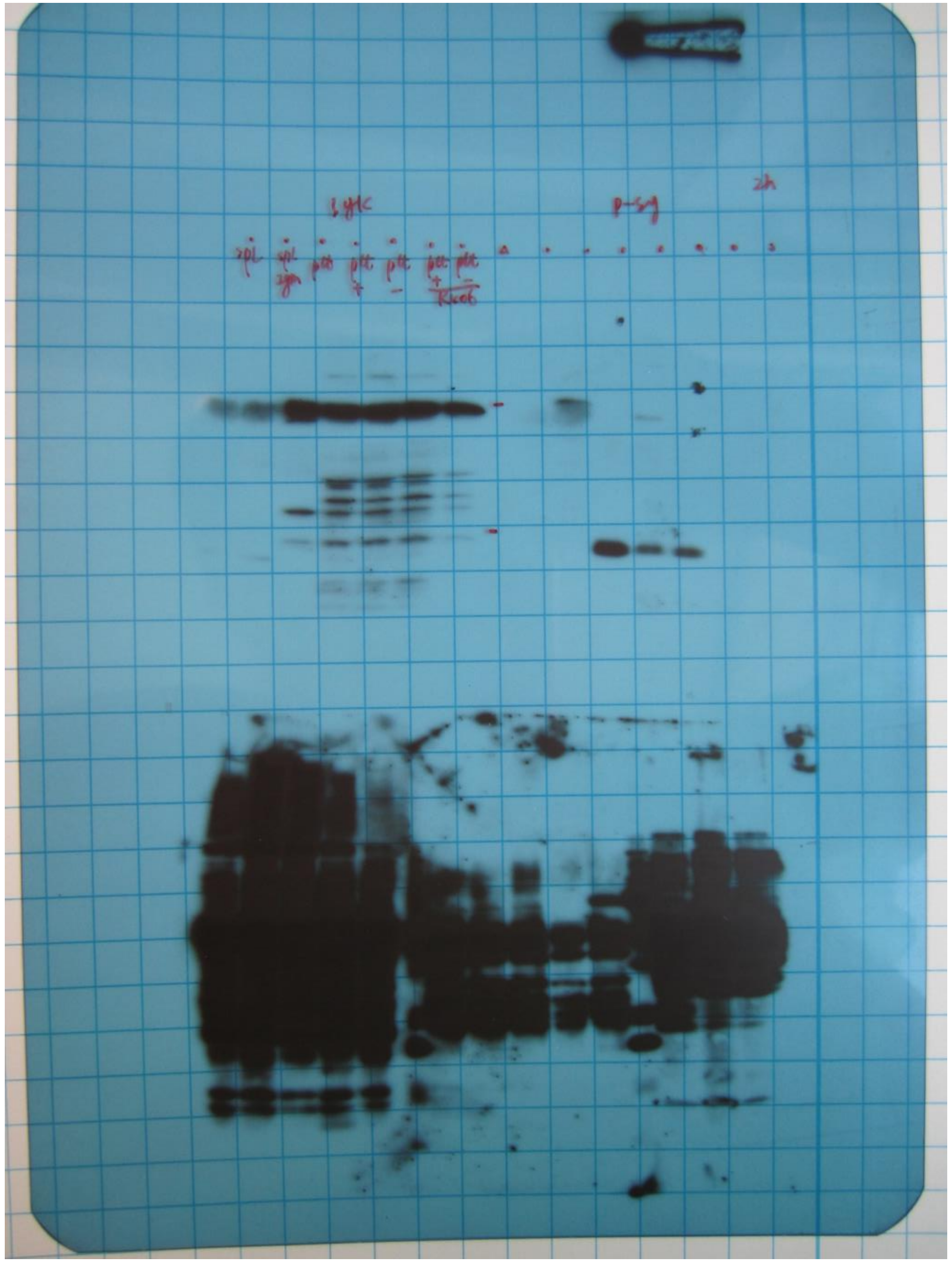

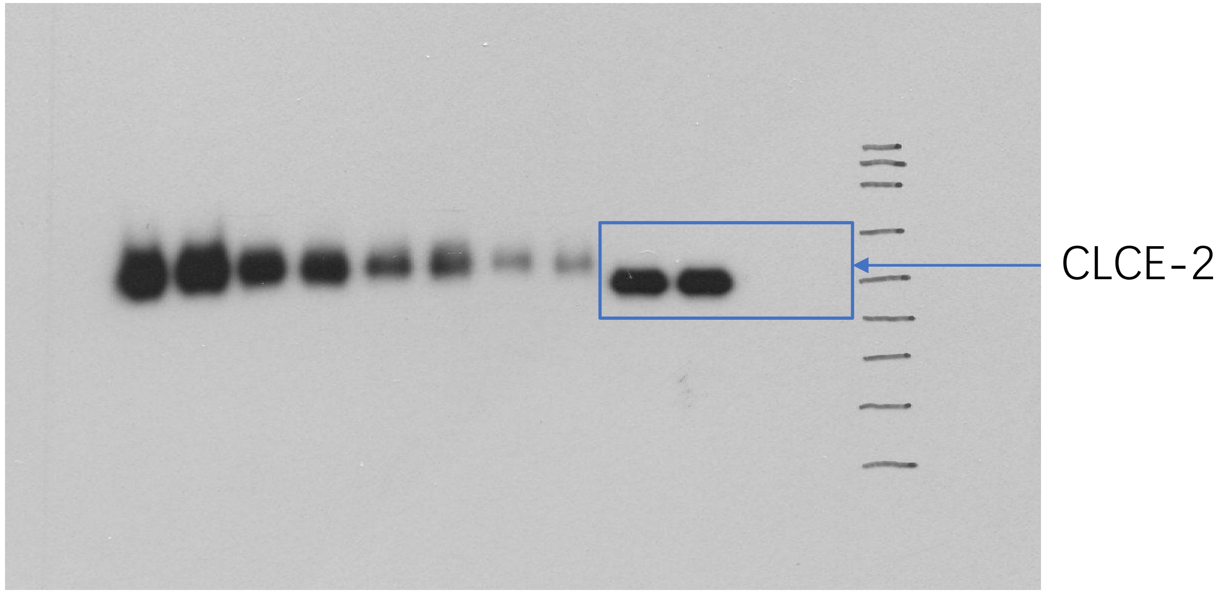


Cropped image of Western blot Film For Supplement Figure 2 Panel B

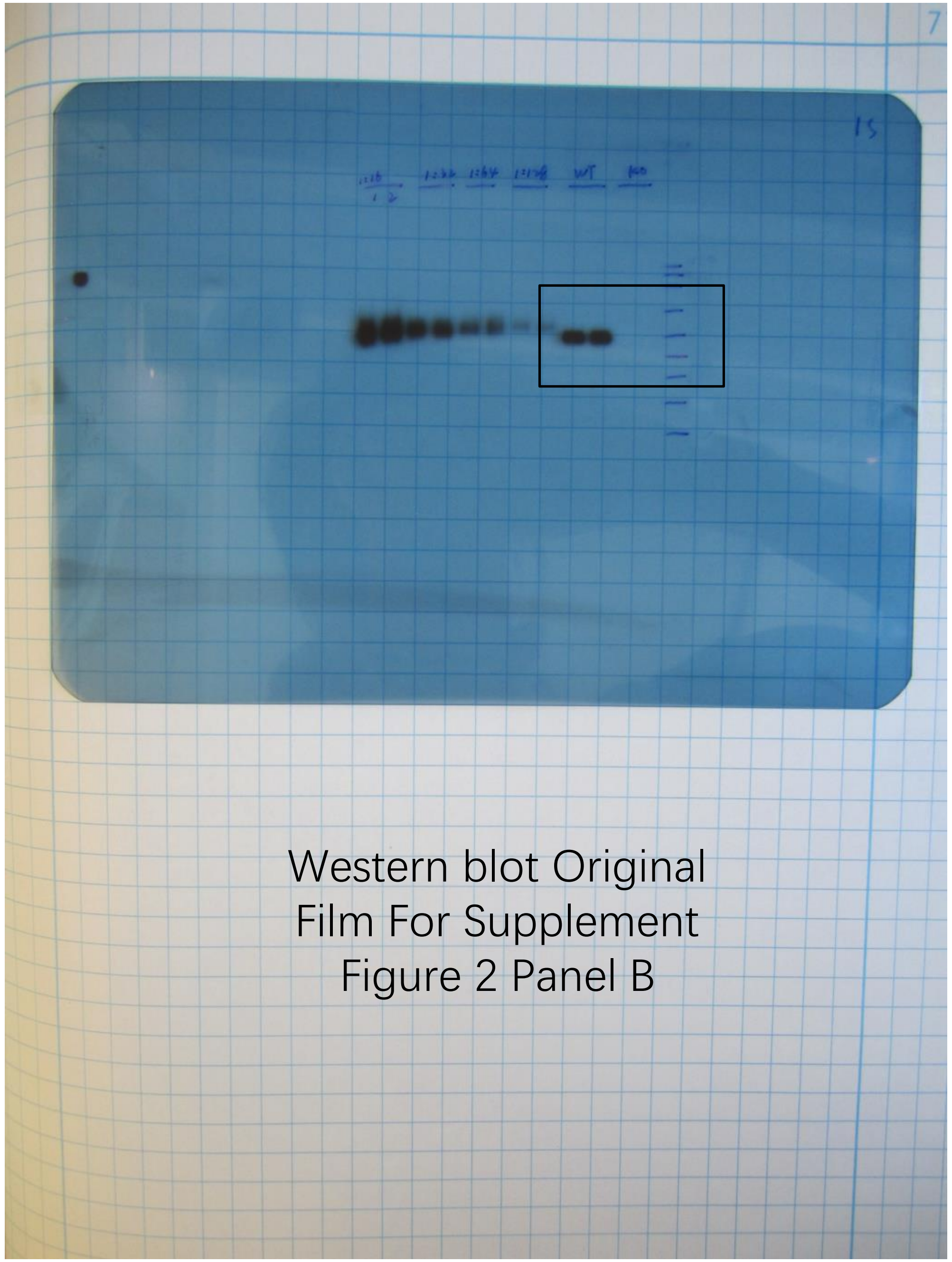

Western blot Original Film For Supplement Figure 2 Panel B

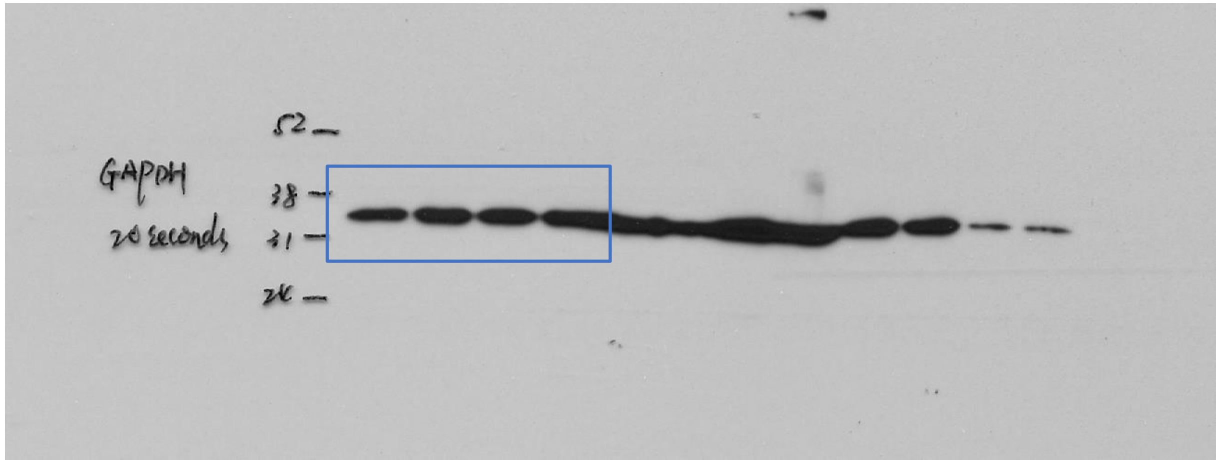


Cropped image of Western blot Film For Supplement Figure 2 Panel B

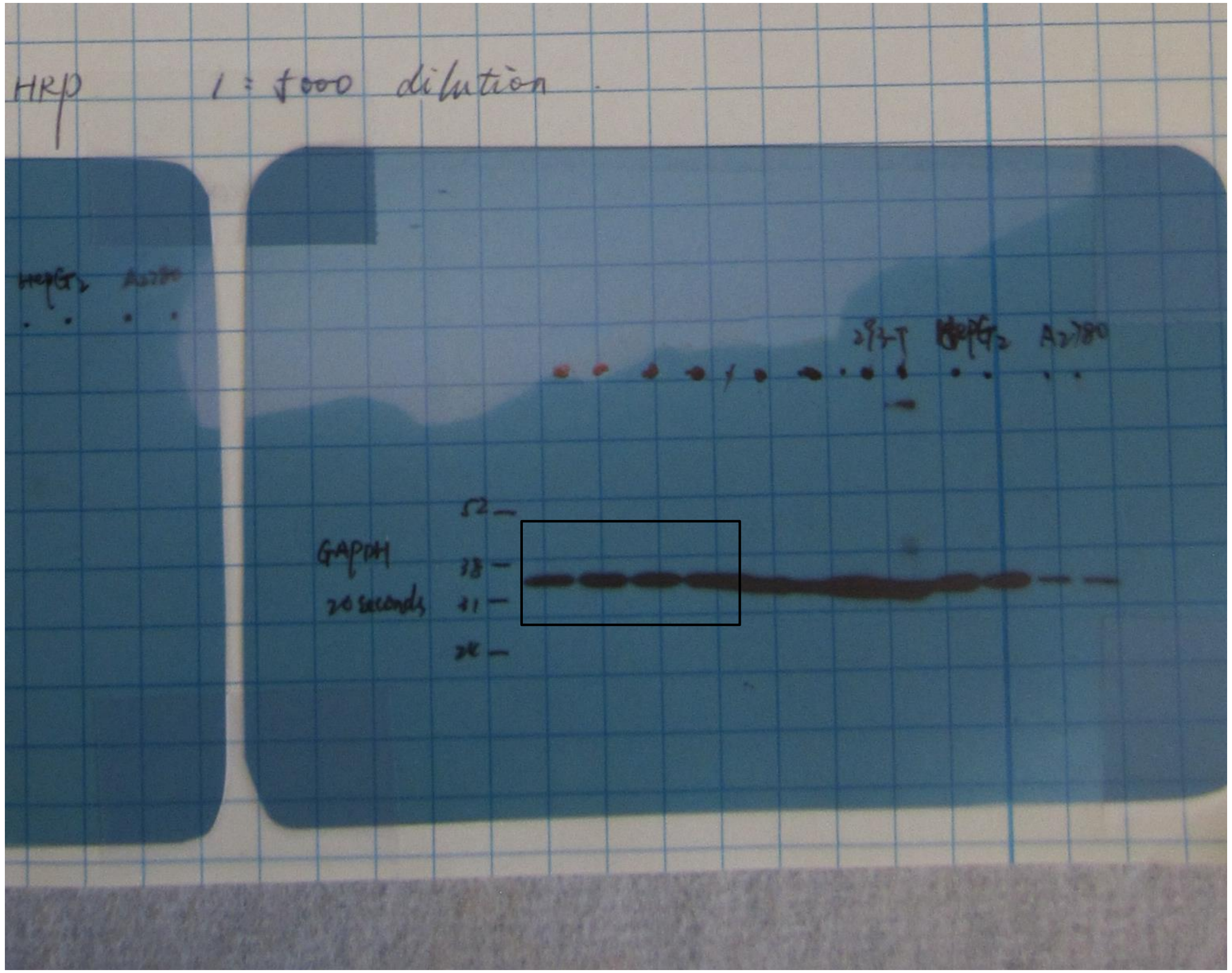


Western blot Original Film For Supplement Figure 2 Panel B
