# Supplementary material for: The podoplanin-CLEC-2 interaction promotes platelet-mediated melanoma pulmonary metastasis
Source: BMC Cancer. 2024 Apr 1;24:399. doi: 10.1186/s12885-024-12194-w (PMC10983743; doi:10.1186/s12885-024-12194-w)
Supplement: Supplementary file 2 — Supplementary Material 2 [file 12885_2024_12194_MOESM2_ESM.pdf]

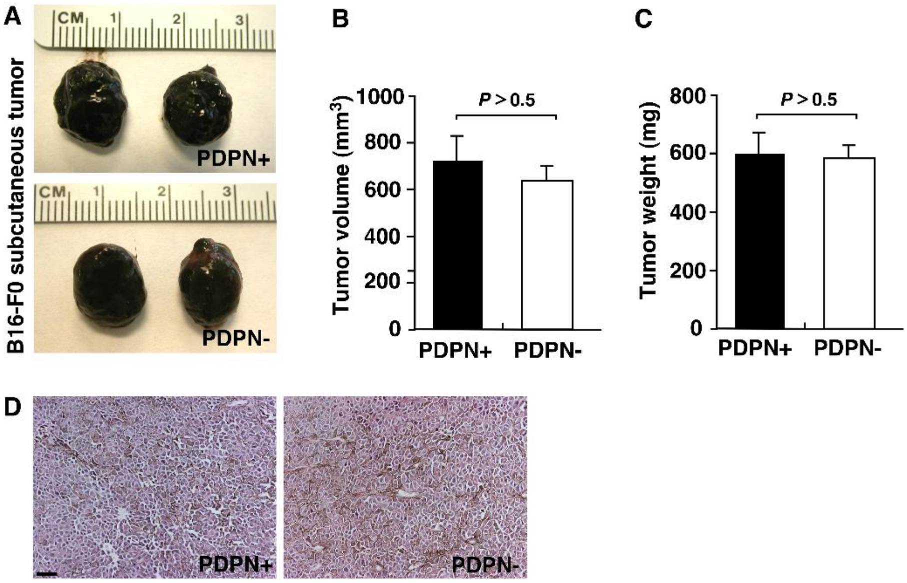

Sup. Figure 1. Podoplanin and CLEC-2 interaction does not confer *in vitro* melanoma cell growth and *in vivo* tumor growth

(A). 2×10^5 B16-F0 PDPN$^+$ and PDPN$^-$ cells were injected subcutaneously into WT C56BL/6J mice respectively. 14 days later, the mice were sacrificed and the tumors were removed. Representative gross images of subcutaneous tumor tissue of each group were shown. **(B).** and **(C).** Tumor volume (volume = 1/2 length × width^2) and tumor weight of each group were measured. Even the volume and weight of PDPN+ cells tumor were higher than PDPN$^-$ cells tumors, but they have no statistical significance (*P>0.5*). **(D).** Representative images of H&E staining of subcutaneous tumor tissue section. The histology of PDPN$^+$ cell injection tumor tissue is similar with that of PDPN- cells injection tumor tissue.

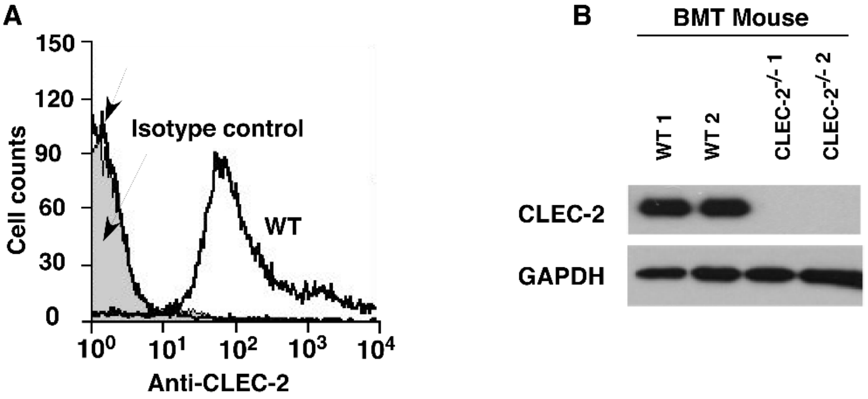

Sup. Figure 2. CLEC-2 deplete efficiency in CLEC-2 deficient mouse

(A). Flow cytometry profile shows CLEC-2 delete efficiency in CLEC-2 KO mouse and WT mouse after bone marrow transplantation. Whole blood from BMT CLEC-2 KO mice and WT mice 4 weeks after bone marrow transplantation were incubated with Biotinylated anti-CLEC1B mAb and treptavidin-PE. Biotinylated rat IgG 2b was used as isotype control. **(B).** Western blot detection of CLEC-2 expression in the cell lysates of Wild type mouse and CLEC-2 deletion mouse.
